# Dedifferentiated Liposarcoma of the Kidney: Surgical Management and Diagnostic Insights

**DOI:** 10.7759/cureus.89512

**Published:** 2025-08-06

**Authors:** Jawad Khan, Amna Kashif, Fatima Safdar, Pir Sameeullah Shah Rashdi, Sadaqat Ullah Rehmat, Hira Shehzad

**Affiliations:** 1 Department of Urology, Ayub Teaching Hospital, Abbottabad, PAK; 2 Department of Surgery, Jinnah Sindh Medical University, Karachi, PAK; 3 Department of Pathology and Laboratory Medicine, Aga Khan University Hospital, Karachi, PAK; 4 Department of Trauma and Orthopaedics, St George's Hospital, London, GBR; 5 Department of Surgery, Ayub Medical College, Abbottabad, PAK

**Keywords:** case report, dedifferentiated liposarcoma, kidney tumor, surgery, tumor management

## Abstract

This report presents the case of a 62-year-old male who presented with a two-month history of right flank pain and decreased appetite. Clinical evaluation revealed a palpable, non-tender mass in the right flank, while laboratory tests demonstrated mild anemia (hemoglobin 9.3 g/dL) with otherwise normal renal function. Contrast-enhanced computed tomography of the abdomen showed a large, heterogeneous mass arising from the lower pole of the right kidney, containing mixed densities, fatty components, and coarse calcifications. The initial differential diagnosis included angiomyolipoma. Following multidisciplinary discussion, the patient underwent radical nephrectomy. Gross examination revealed a lobulated, yellowish tumor with areas of necrosis and hemorrhage. Histopathological analysis confirmed the diagnosis of dedifferentiated liposarcoma, characterized by spindle cell proliferation with moderate nuclear atypia and mature adipocytes. Immunohistochemistry showed strong nuclear positivity for MDM2 and p16, supporting the diagnosis. The tumor was staged as pT3aNxM0. Postoperative follow-up was arranged to monitor for recurrence. This case highlights the diagnostic challenge posed by rare renal tumors with fatty components and emphasizes the importance of including dedifferentiated liposarcoma in the differential diagnosis. Early surgical intervention remains essential to optimize outcomes, given the tumor’s limited response to adjuvant therapies.

## Introduction

Liposarcoma (LPS) is an adipose connective tissue malignancy and is responsible for about 20% of soft tissue sarcomas [1]. Histological types of LPS are well-differentiated, dedifferentiated, myxoid, round cell, and pleomorphic types [2]. Literature sources state that LPS most frequently occurs in the limbs [3]. Dedifferentiated liposarcoma (DDLPS) is of particular importance because it is an aggressive tumor that changes from a low-grade lipogenic tumor to a high-grade non-lipogenic sarcoma [4].

DDLPS infrequently affects kidney function [5], but it poses significant diagnostic challenges due to the resemblance of its radiological features with other renal tumors with high fat content, i.e., angiomyolipoma and renal cell carcinoma of certain types [6]. Although imaging techniques play a vital role in the initial diagnosis, a histopathological examination is necessary to establish a final diagnosis. Use of immunohistochemical markers, e.g., MDM2 and CDK4, is crucial to distinguish DDLPS from other renal lesions.

Complete surgical resection is the primary treatment since the tumors have a poor response to chemotherapy and radiotherapy [7]. The present article reports a rare primary renal DDLPS case with the diagnostic techniques employed and imaging characteristics utilized, and the adopted treatment protocol.

## Case presentation

This study presents the case of a 62-year-old male patient who presented with chief complaints of right flank pain and decreased appetite for two months, accompanied by nausea and decreased appetite. Physical examination revealed a firm, non-tender, palpable mass in the right flank. Vital signs were within normal limits.

Laboratory investigations revealed mild anemia with a hemoglobin level of 9.2 g/dL, while white blood cell count and renal function tests were normal (Table 1).

**Table 1:** Laboratory results

Test	Result	Reference	Unit
White blood cells	8.1	4-11	cells/µL
Hemoglobin	9.2	11.5-17.5	g/dL
Urea	28.2	18-45	mg/dL
Creatinine	0.9	0.7-1.4	mg/dL

Abdominal ultrasound demonstrated a large, heterogeneous, predominantly hyperechoic mass arising from the lower pole of the right kidney with cystic changes.

**Figure 1:**
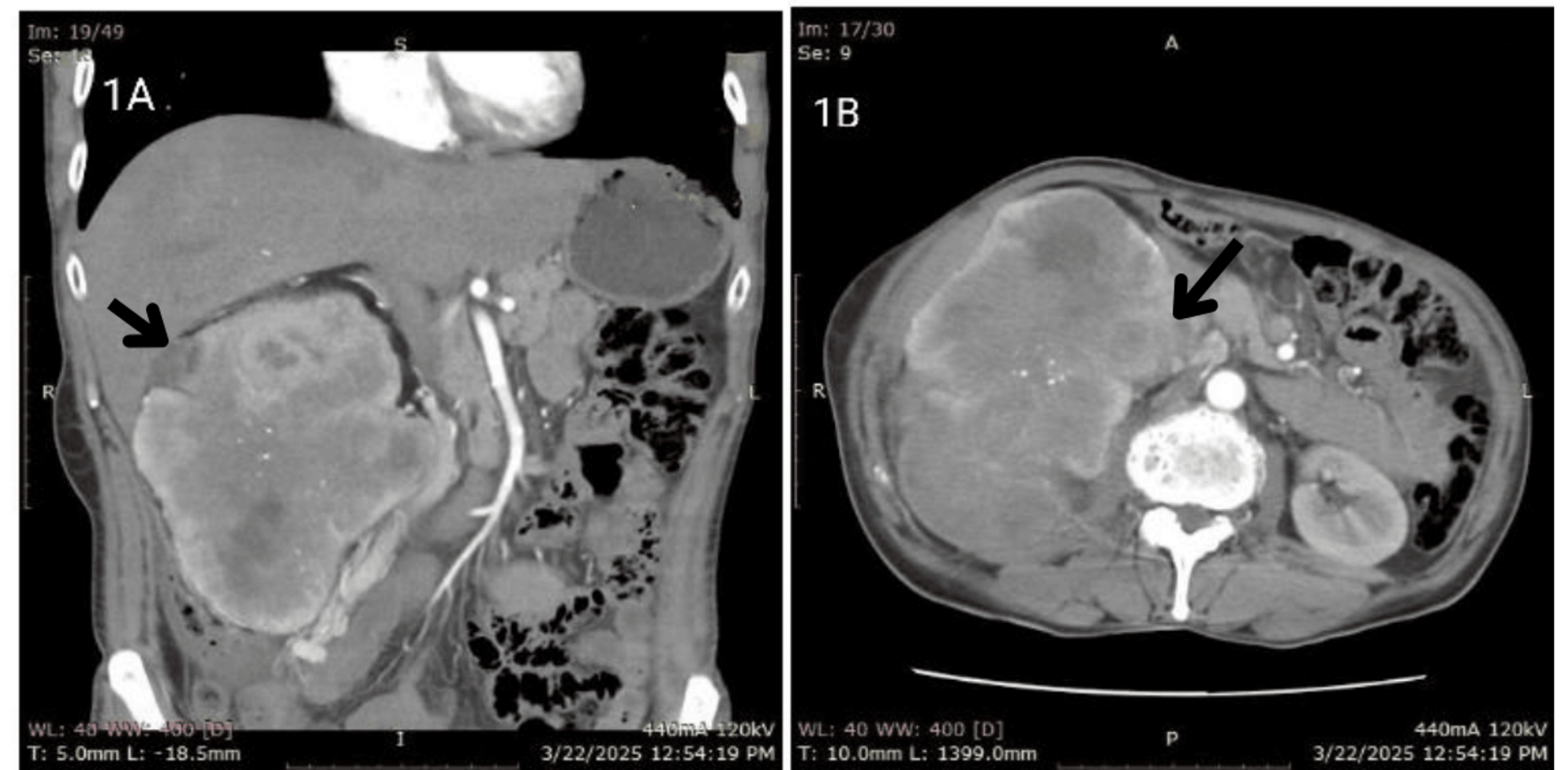
CECT abdomen and pelvis showing a large, well-defined heterogeneous mass originating from the lower pole of the right kidney exhibiting mixed density with fatty components, cystic degeneration, and coarse calcifications (1A) Axial view; (1B) coronal view. CECT, contrast-enhanced computed tomography.

Contrast-enhanced computed tomography (CECT) of the abdomen and pelvis demonstrated a large, well-defined heterogeneous mass of 15*10*8 cm in size originating from the lower pole of the right kidney, exhibiting mixed density with fatty components, cystic degeneration, and coarse calcifications (Figure 1A, 1B).

The patient was discussed in a multidisciplinary team (MDT) meeting, and a right open radical nephrectomy was performed. Intraoperatively, the mass displayed dense adhesions to the adjacent liver and bowel loops, but no gross invasion was noted.

**Figure 2:**
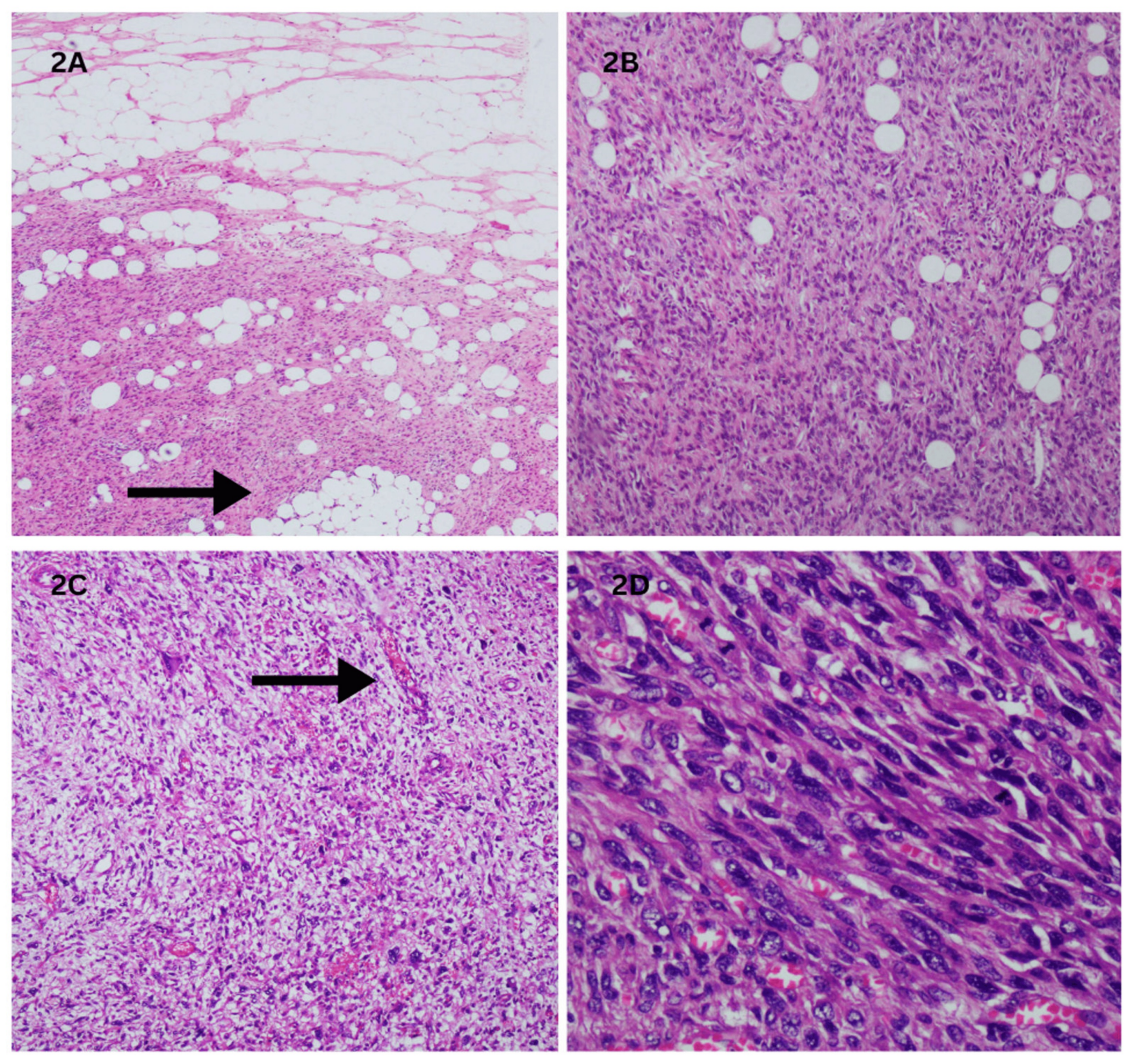
Histopathology showing infiltrating neoplastic lesion (2A) Low-power view showing an infiltrating neoplastic lesion predominantly composed of spindle cells with moderate atypia and admixed lobules of mature adipocytes (marked by arrow) (H&E, ×10). (2B) High-power view of the same lesion showing pleomorphic enlarged spindle to ovoid nuclei with vesicular chromatin and prominent nucleoli with scant eosinophilic cytoplasm (H&E, ×20). (2C) Stroma exhibits thin-walled vessels (marked by arrow) (H&E, ×20). (2D) High-power view showing marked nuclear pleomorphism and scattered lipoblasts (H&E, ×40).

Gross inspection of the removed specimen revealed a large, multi-lobulated, yellow mass containing areas of necrosis and hemorrhage. Microscopic evaluation demonstrated an invasive neoplasm mainly composed of spindle-shaped cells with moderate atypical features, intermingled with clusters of mature adipocytes (Figure 2A, 2B). The stroma exhibited thin-walled vessels, and the cells demonstrated pleomorphic enlarged spindle to ovoid nuclei with vesicular chromatin and prominent nucleoli (Figure 2C, 2D).

**Figure 3:**
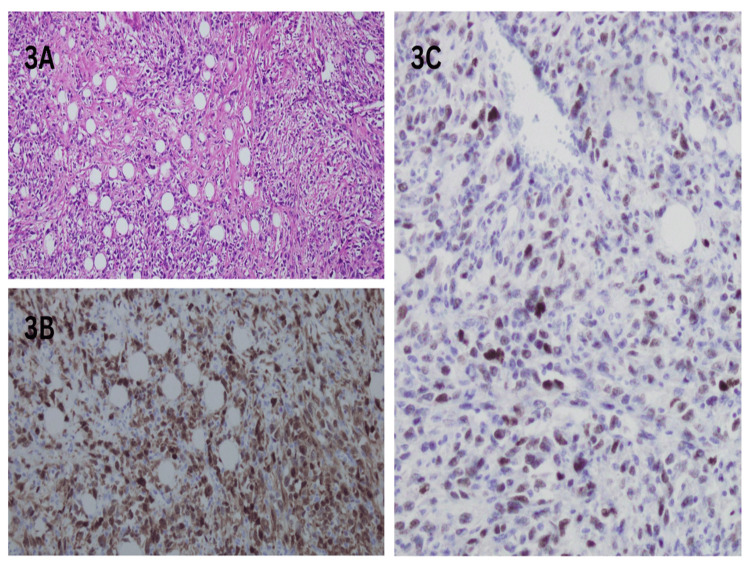
Immunohistochemical staining shows spindle cells atypia and p16 and MDM2 positivity (3A) High-power view showing spindle cells with moderate to marked atypia and admixed lipoblasts (H&E, ×40 magnification). (3B) Immunohistochemical stain p16 is nuclear positive in neoplastic cells (×40 magnification). (3C) Immunohistochemical stain MDM2 is nuclear positive in neoplastic cells (×40 magnification).

High-power examination revealed spindle cells with moderate to marked atypia and admixed lipoblasts (Figure 3A). Immunohistochemical staining demonstrated strong nuclear positivity for p16 and MDM2 in the neoplastic cells (Figure 3B, 3C).

The final pathological stage was pT3aNxM0.

The case was discussed once more in the MDT meeting, and it was decided to repeat the CECT of the abdomen and pelvis after three months and then every six months for two years. If no recurrence is detected within two years, the case will be reviewed again in the MDT, and a further follow-up plan will be determined.

## Discussion

The term "dedifferentiation" was first introduced in the medical literature in 1971 to describe the progression of low-grade sarcomas to high-grade sarcomas [8]. DDLPS accounts for approximately 10-20% of all LPS and is typically characterized by the transition from well-differentiated adipocytic areas to high-grade non-lipogenic sarcoma [9]. While DDLPS commonly arises in the retroperitoneum, extremities, and paratesticular region, primary renal involvement is exceedingly rare [5].

Clinically, patients with renal DDLPS may present with non-specific symptoms such as flank pain or a palpable mass [10]. Imaging studies of retroperitoneal or renal DDLPS often reveal a large, heterogeneous mass with fat and soft-tissue components, occasionally with calcifications. These features can overlap with other fat-containing tumors, such as renal angiomyolipoma, posing a diagnostic challenge [6]. Therefore, definitive diagnosis requires histopathological examination, including immunohistochemistry.

Histopathologically, DDLPS is characterized by a biphasic pattern with areas of well-differentiated adipocytic tissue admixed with high-grade spindle cell sarcoma (Figure 2A, 2B). The presence of pleomorphic spindle cells with vesicular chromatin and prominent nucleoli, along with thin-walled vessels, is characteristic (Figure 2C, 2D). Immunohistochemical staining with markers such as p16 and MDM2 helps differentiate DDLPS from other renal neoplasms (Figure 3B, 3C).

MDM2 and CDK4 are commonly overexpressed in DDLPS due to amplification of the 12q13-15 chromosomal region [2]. Immunohistochemical staining for these markers is highly sensitive and aids in differentiating DDLPS from other renal neoplasms [2]. In the present case, diffuse nuclear positivity for MDM2 and p16 confirmed the diagnosis.

Surgical resection with negative margins remains the cornerstone of DDLPS treatment, as the tumor is relatively resistant to chemotherapy and radiotherapy [7]. Complete resection reduces the risk of local recurrence [7]. Postoperative surveillance with periodic imaging is essential due to the risk of recurrence and metastasis [6].

## Conclusions

This case highlights the importance of including DDLPS in the differential diagnosis of large, heterogeneous renal masses. Histopathological examination, including immunohistochemistry, is essential for accurate diagnosis. Early surgical intervention and vigilant follow-up are critical for optimal patient outcomes.
